# Ultra-Sensitive Fluorimetric Method for the First Estimation of Vonoprazan in Real Human Plasma and Content Uniformity Test

**DOI:** 10.1007/s10895-022-02979-2

**Published:** 2022-06-07

**Authors:** Roshdy E. Saraya, Yasser F. Hassan, Walid E. Eltukhi, Baher I. Salman

**Affiliations:** 1 Pharmaceutical Analytical Chemistry Department, Faculty of Pharmacy, Port Said University, Port Said, 42511 Egypt; 2 Pharmaceutical Analytical Chemistry Department, Faculty of Pharmacy, Al-Azhar University-Assiut Branch, Assiut, 71524 Egypt

**Keywords:** Vonoprazan, Content uniformity, Pharmacokinetics, NBD-Cl

## Abstract

Vonoprazan (VON) has been approved recently via US-FDA in 2015 as the first in class of potassium competitive acid blocker group. VON is used for management of GIT ulcer, reflux esophagitis and for eradication of Helicobacter pylori. So, the first spectrofluorimetric method was developed for estimation of VON in real human plasma and content uniformity test. The fluorimetric methodology based on reaction of secondary amine group in VON with benzofurazan (0.05% w/v NBD-Cl) reagent as nucleophilic substitution reaction in alkaline medium (0.1 M borate buffer pH 8.2) to produce highly fluorescent product measure at 530 nm after excitation at 465 nm. The linear calibration range was found 15 to 200 ng mL^−1^ with lower limit of quantitation (LOQ) equal to 8.57 ng mL^−1^. The method was successfully applied for estimation of VON in pharmacokinetic (PK) and content uniformity studies. The maximum plasma concentration was found to be (C_max_) 71.03 ng mL^−1^ after maximum time (t_max_) equal to 1.5 ± 0.15 h. The presented strategy also applied to ensure concentration of drug in each tablet using content uniformity test with high percent of recovery 100.05 ± 0.66. The proposed method was established for clinical laboratories and therapeutic drug monitoring studies.

**Supplementary Information:**

The online version contains supplementary material available at 10.1007/s10895-022-02979-2.

## Introduction

**Fig. 1:**
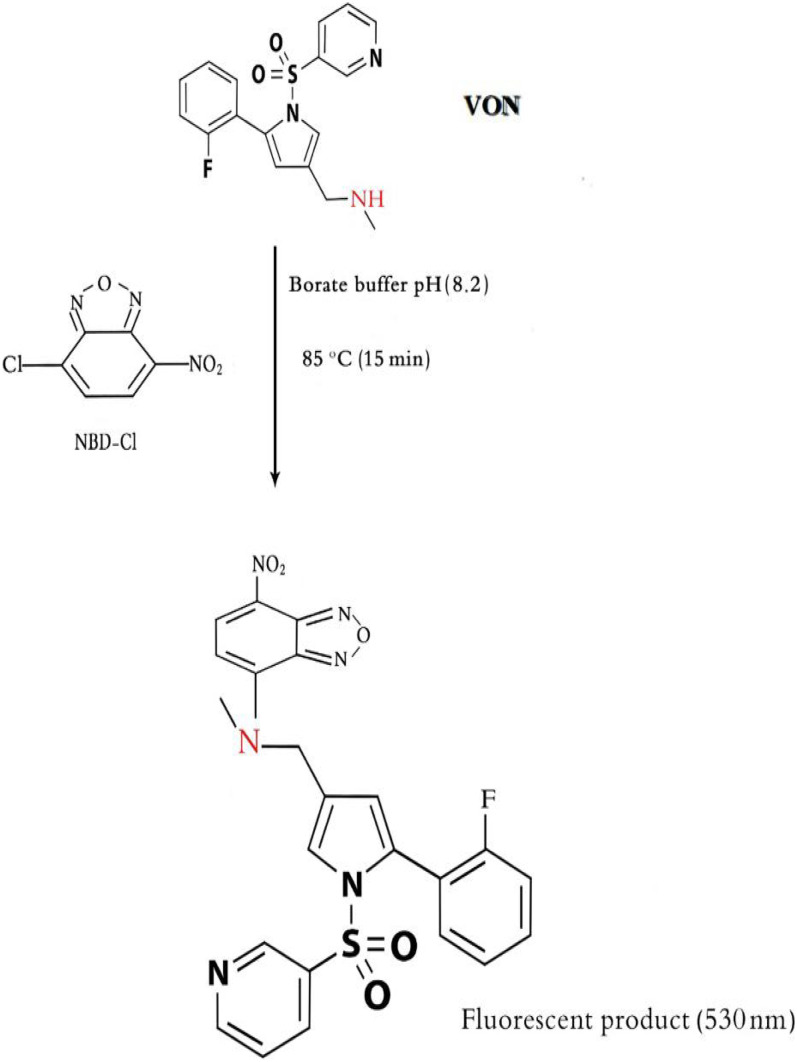
The proposed reaction mechanism between VON and NBD-Cl

VON (Fig. 1), IUPAC name: 1-(5-(2-fluorophenyl)-1-(pyridin-3- ylsulfonyl)-1H–pyrrol − 3-yl)-N-methylmethanamine fumarate. VON is the first drug as potassium competitive acid blocker group used for management GIT ulcer and esophageal reflux [1]. In last few years, the fluorimetric approach is the most applicable approach due to high sensitivity, simplicity, economical, easy handling in clinical laboratories and its selectivity for estimation of varying pharmaceutical products [2–4]. Few analytical methods have been reported for estimation of VON as high performance liquid chromatography (HPLC) [5, 6] and electrochemical method [7]. Some drawbacks were reported in these methods as using expensive equipment’s, reagents, time consuming, and lack to sensitivity. The presented work is the first spectrofluorimetric method for estimation of VON in human plasma and content uniformity test with high sensitivity. 7-chloro-4-nitrobenz-2-oxa-1,3-diazole is neucliphic substitution reagent used for fluorimetric derivatization reactions with amine groups (primary and secondary) to produce highly fluorogenic product in alkaline medium [2, 8–11].

The aim of this study is to develop ultra-sensitive, selective, and economical fluorimetric method. The method was successfully applied to a pharmacokinetic study in healthy human volunteers and content uniformity test in tablet dosage form. The pharmacokinetic parameters were studied as maximum plasma concentration, half-life and maximum time for absorption which make the presented method easily used for therapeutic drug monitoring system.

## Experimental Section

### Instruments

The spectrofluorimetric measurements were done using Scinco (FS-2 fluorimeter, Scinco, Korea), with 1 cm quartz cell, excitation and emission slit width set at 5 nm and photo multiplied tube (PMT) voltage 400 V). Water bath (WB-1R1H-3 thermostatic) China, Benchtop pH meter (China), high speed centrifuge (China), MS-TS analytical digital balance (USA). MATLAP software for PK analysis.

### Chemical and Solutions

Vonoprazan (VON 99.89%) and vonaspire® tablets (20 mg/tablet) was obtained from obtained from Alrowad pharmaceutics, Egypt. 4-Chloro-7-nitrobenzofurazan (NBD-Cl) was obtained from Sigma Aldrich (USA). Methanol, ethanol, acetonitrile, boric acid, acetone, borax, sodium hydroxide and hydrochloric acid were obtained from El-Gomhoria Co, Assiut, Egypt. Human plasma samples were achieved from 6 healthy human volunteers following ethical standards of the responsible committee and kept at -10 degree.

### Preparation of Solutions

Stock solution of VON was prepared using 5 mg of authentic powder dissolved in ultra-pure water 50 mL into 100 mL-volumetric flask. The solution was sonicated for 3 mint to ensure the complete solubility of VON drug then completed to the mark with the same solvent. Further dilutions with distilled water were performed to prepare working solution in a concentration range from (150 – 2000 ng mL^−1^).

Borate buffer (0.1 M) was prepared [9, 10] and adjusted to pH range using 0.1 M NaOH and 0.1 M HCl.

NBD-Cl (0.05% w/v*)* reagent prepared by 5 mg powder was dissolved using 50 mL methanol into a 100 mL volumetric flask, then completed with methanol to the mark.

### Preparation of Samples

#### Biological Samples

Plasma samples were collected and carried out according to (ethical standards of the responsible committee on human experimentation) (institutional and national) and with Helsinki Declaration of 1975, as revised in 2008.

#### Spiked Plasma Samples

Five milliliters of the blood samples were collected from 6 healthy human volunteers (males and females aged from 30 to 40 years) into 10 mL screw-capped heparinized tube. The tubes were centrifuged at 5000 rpm for 25 min to separate the plasma. The plasma samples (300 µL) were spiked with different concentrations of working solution of VON. Two milliliters of methanol were added as protein precipitating agent. The resultant solution was completed to 10 mL with ultra-pure water and then centrifuged at 4000 rpm for 20 min. One milliliter from the supernatant was used at general procedures.

#### Pharmacokinetic Study

*The blood samples* were collected from 6 healthy human (males and females aged from 30−40 years) volunteers after administration vonaspire® tablets (40 mg/tablet VON) as a single oral dose. After that, serial venous blood samples (5 mL) were collected in heparinized tubes for 24 h after dosing (0 h) and after 0.25, 0.5, 0.75, 1, 1.5, 2, 3, 4, 6, 10, 15, 20 and 24 h). The tubes were centrifuged at 5000 rpm for 25 min to separate the plasma. The plasma samples frozen at -20 until analysis. Then the samples treated as spiked method without addition of the drug.

### Pharmaceutical Dosage Form

Ten vonaspire ® tablets (40 mg VON/tablet) were accurately weighed, finely powdered and mixed. An accurately weighed amount of the powdered tablets equivalent to 10 mg of VON was transferred into 100-mL volumetric flask followed by addition of 60 mL of ultra-pure water. The solution was sonicated for 5 min then followed by filtration and dilution with ultra-pure water to provide final concentration of 100 µg mL^−1^. A series of working standard solutions with concentration range between 150 ng mL^−1^- 2000 ng mL^−1^ were prepared in volumetric flasks.

*The content uniformity* testing was assessed and performed through analysis of ten tablets individually according to official USP guidelines [12]. Each tablet was individually weighed, finely powdered, and analyzed using the fluorescence procedure.

### Fluorescence Procedure

One milliliter from VON working solution (150 – 2000 ng mL^−1^) was transferred into a series of 10-mL stoppered autoclaved test tubes, 1.0 mL of 0.1 M borate buffer (pH 8.2) was added followed by 0.5 mL of NBD-Cl (0.05% w/v). The tubes were heated in water bath at 85 °C for 15 min. The tubes were rapidly cooled in ice water bath and 0.75 mL from 2 M HCl solution was added. The solution quantitatively transferred to 10-mL calibrated flask and completed to the mark with methanol in order to provide final concentration range (15 – 200 ng mL^−1^). The fluorescent product was measured at 530 nm (λex at 465 nm). The blank experiment was performed similarly omitting the drug.

### Stoichiometry Mechanism

The Stoichiometry of the reaction was studied using equimolar concentration (1 × 10^–3^ M) from VON and NBD-Cl (0.05% w/v) by application continuous variation method [2, 9, 13]. A series of solutions where portions of mixture of master solutions of VON and NBD-Cl were made up as (0.1: 0.9, 02: 0.8, 0.3: 0.7, 0.4: 0.6, 0.5: 0.5, 0.6: 0.4, 0.7: 0.3, 0.8: 0.2 and 0.9: 0.1, respectively). The fluorescence procedure was followed.

## Results and Discussion Part

**Fig. 2:**
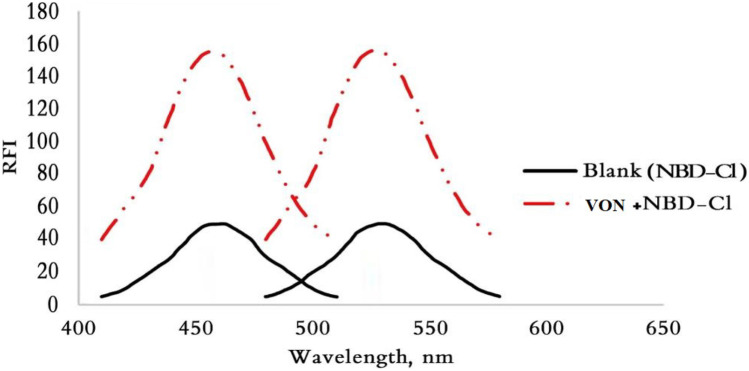
Excitation and emission spectra for reaction of VON (100 ng mL.^−1^) with NBD-Cl

NBD-Cl is nucleophilic substitution reagent reacts with primary and secondry amine groups producing highly fluorescent product in alkaline medium [9, 10, 13]. However, some drawbacks were reported in published method as long heating time (55 min) which consider time consuming [13, 14], high concentration of the reagent [11, 13, 15]. While, the presented study provides sensitive, time saving and economical method for analysis of VON in human plasma which applied in pharmacokinetic study and content uniformity test. In the developed work, the reaction of secondary amine moiety in VON structure with NBD-Cl (0.05%w/v) was achieved using borate buffer at pH 8.2 ± 0.2 and heating at 85 °C for only 15 min as in Fig. 1 with stoichiometry of the reaction 1:1. The fluorogenic product measure at 530 nm after excitation at 465 nm (Fig. 2).

### Optimization of the Methodology

The fluorescence reaction between VON and NBD-Cl is based on different factors as; pH, temperature, heating time, NBD-Cl concentrations, and diluting solvents.

### Effect of Buffer System and pH

**Fig. 3:**
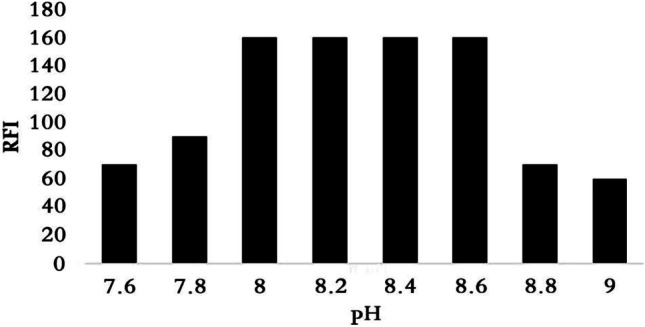
Effect of pH range (0.1 M borate buffer) on the reaction of VON (100 ng mL.^−1^) with NBD-Cl (0.05% w/v)

The nucleophilic substitution reaction between VON and NBD-Cl reagent for formation highly fluorescent product is pH dependent. Hence, different type of buffers was studied as (Teorell, borate, phosphate and BR) as shown in Fig. S1 (supplementary Materials). The maximum fluorescent intensity was obtained using 0.1 M borate buffer (pH 8.2 ± 0.3) (Fig. 3).

Using borate buffer at pH more than pH 8.6 led to decrease in the fluorescence intensity due to the increase in hydroxide ions concentration which holds back the reaction between VON and NBD-Cl and this agrees with previously reported studies [2, 9, 10, 15]. Moreover, the effect of buffer volume on the fluorescence intensity was studied, where different volumes from 0.25 to 3 mL were investigated. It was found that maximum RFI obtained using 1.0 ± 0.25 mL of borate buffer pH 8.2 (Fig. S2).

### Effect of NBD-Cl Concertation

**Fig. 4:**
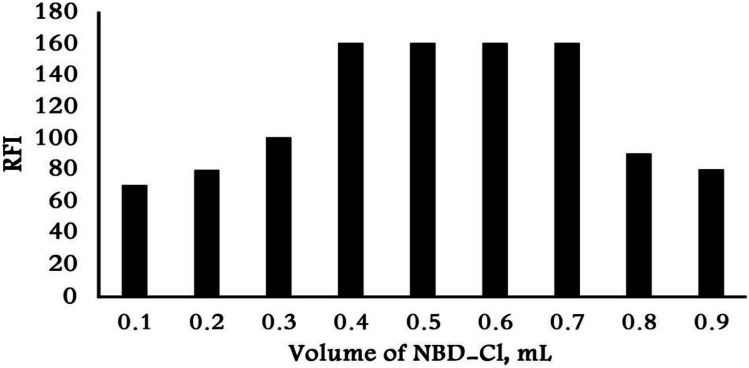
Effect of volume on NBD-Cl on the reaction with VON (100 ng mL.^−1^)

Varying volumes of NBD-Cl reagent (0.1 to 0.9 mL) were utilized to study the effect of the reagent. As shown in Fig. 4 the maximum RFI was achieved via applying 0.5 mL of (0.05% w/v) NBD-Cl, after trying varying volumes of NBD-Cl reagent. Increasing the volume of NBD-Cl than 0.7 mL led to decrease RFI and increasing of the blank intensity. So, 0.5 mL of (0.05% w/v) NBD-Cl was used as the optimum condition.

### Effect of Temperature and Heating Time Effect

**Fig. 5:**
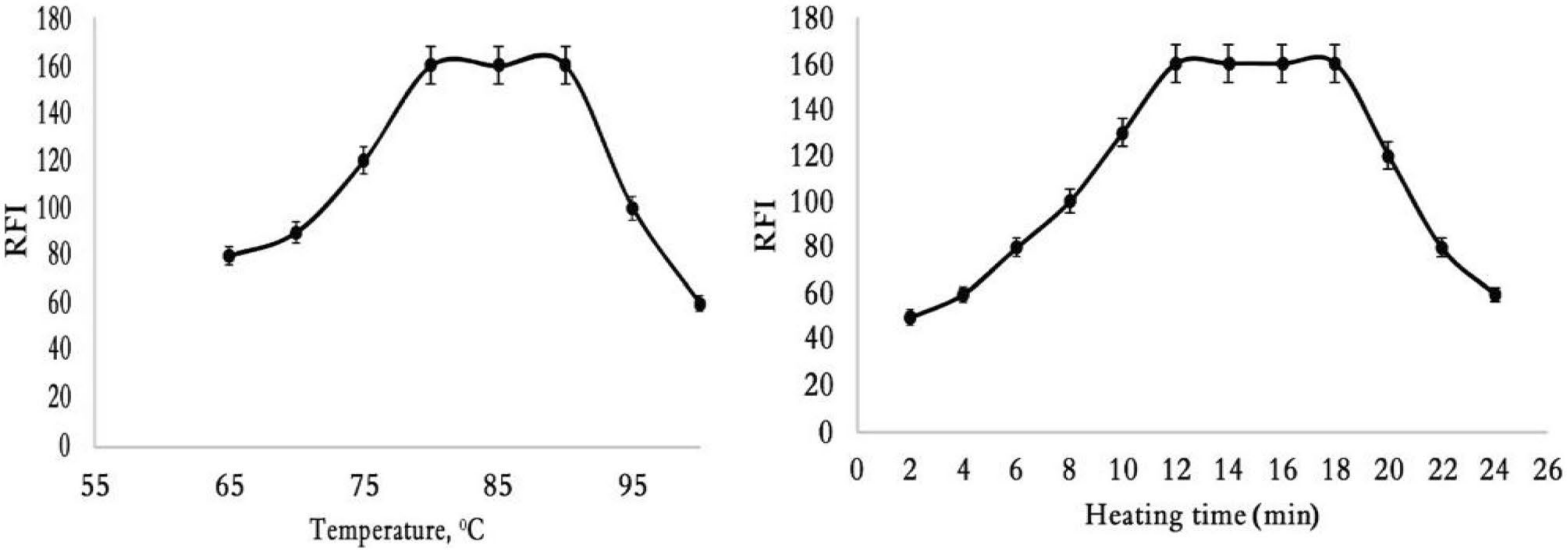
Effect of temperature and heating time for reaction of VON (100 ng mL.^−1^) with NBD-Cl (0.05% w/v)

The nucleophilic substitution reaction between secondary amine moiety in VON and Cl^−^ in NBD-Cl is based on temperature and heating time. As seen in Fig. 5, varying temperature degrees were studied under time intervals, the maximum RFI was obtained using 85 ± 5 °C for 15 min.

### Effect of HCl Concentration

Acidification of the reaction media between VON and NBD-Cl is required before the measuring of results because formation of hydroxy-7-nitrobenzo-2-oxa-1,3-diazole bi- product (NBD-OH) [2, 9, 10]. The NBD-OH was quenched using 0.75 ± 0.25 mL from 2 M HCl without any effect on the reaction results (Fig. S3).

### Effect of Diluting Solvents

The influence of diluting solvents as distilled water, methanol, ethanol, acetonitrile, acetone and DMF was studied. Methanol was observed to be the best diluting solvent as it gave the highest fluorescence with good reproducibility and low blank readings.

## Validation of the Methodology

### Linearity Range and Sensitivity

The calibration range of the reaction was achieved by plotting concentration of VON versus RFI. The linear equation was found y = 1.5401x + 3.103 with standard deviation (SD) equal to 1.31. the linear range was found to be 15 – 200 ng mL^−1^ with LOQ equal to 8.5 ng mL^−1^ which indicated to the ultra-sensitivity of the fluorimetric method as in Table 1. Lower limit of quantitation and detection were calculated using International Council for Harmonization guidelines [16] using following equations: LOQ = 10 ϭ/ slope and LOD = 3.3 ϭ/ slope. (ϭ) is the standard deviation.

**Table 1:** Quantitative analytical parameters for estimation of VON using the proposed method

**Parameters**	**VON**
**λex (nm)**	465
**λem(nm)**	530
**Concentration range (ng mL**^**−1**^**)**	15 – 200
**Correlation coefficient (r)**	0.9997
**Determination coefficient (r**^**2**^**)**	0.9998
**Slope**	1.54
**Intercept**	3.10
**SD the intercept (Sa)**	1.31
*LOD ( ng mL^**−1**^**)**	2.80
**LOQ (ng mL^**−1**^**)**	8.50

^*^LOD Limit of detection

^**^LOQ Limit of quantitation

### Accuracy and Precession

Five concentrations within the calibration range were used for studying the accuracy of the proposed method (20,30, 50, 100, 150 ng mL^−1^) as seen in Table 2. The results refer to high accuracy of the proposed method with high percent of recovery ranged from 99.53 to 101.62%.

**Table 2:** Accuracy of the proposed method for estimation of VON using NBD-Cl reagent

**Sample number**	**VON**			
	**Taken** **(ng mL**^**−1**^**)**	**Found** **(ng mL**^**−1**^**)**	% Recovery^*^	**% RSD**
**1**	20	19.94	99.70	0.61
**2**	30	29.86	99.53	0.94
**3**	50	50.20	100.04	1.09
**4**	100	100.16	100.16	0.98
**5**	150	152.44	101.62	0.81

^*^Average of five determinations

Besides, 3 concentrations were applied for intraday and inter-day precession study. The results refer to excellent precision of the proposed method with high percent of recovery 101.43 ± 0.34.

In addition, bio-analytical validation of the reaction between VON and NBD-Cl was studied using six concentrations according to US-FDA recommendations [17]. According to the results in Table 3, the results refer to high accuracy and precision of the proposed method in human plasma.

**Table 3:** Accuracy and precision of the fluorimetric reaction in human plasma

**Intra-day assay(n = 6)**			**Inter-day assay(n = 18)**	
**Conc. (ng mL**^**−1**^**)**	**Accuracy (%)**	**Precision (% RSD)**	**Accuracy (%)**	**Precision (% RSD)**
15	97.33	1.18	97.00	0.98
20	97.15	1.55	96.94	1.08
60	97.00	1.30	96.94	1.90
100	98.09	1.15	97.60	1.11
150	96.21	1.76	96.08	1.46
200	97.38	1.32	96.88	1.80

### Robustness

The robustness of this study was checked by applying minor changes on parameters of analytical procedure. The results in table (Table S1) there was no significant effect for small change in method variables.

### Incurred Sample Reanalysis (ISR)

Further incurred sample reanalysis (IRS) was carried out to evaluate accuracy and precision of incurred sample using FDA guidelines. The difference values between initial samples and incurred samples do not exceed than 1.82 to 4.03% (Table S2).

### Selectivity of the Proposed Method

The method selectivity was checked to observe any interference from excipient. VON was studied in the presence of varying different excipient as talc 10 mg, starch 100 mg, mannitol 10 mg, magnesium stearate 10 mg, lactose 10 mg, and sodium chloride 10 mg. The results refer to there was no interference from the excipients which refer to the high selectivity of the proposed methods (Table S3).

### Stability of VON in Human Plasma

Varying parameters were applied to check the stability of VON in plasma samples as three Freeze–thaw cycle stability (-24 °C), long-term stability (1 month at -24 °C), short-term stability (12 h at -24 °C), post-preparative stability (6 h at room temperature 25 °C), post-preparative stability (12 h at room temperature 25 °C) using three concentrations, low quality control (LQC 20 ng mL^−1^), medium quality (MQC 100 ng mL^−1^) and high quality control (HQC 150 ng mL^−1^) levels. The percentage of recoveries compared with freshly prepared samples between 95.11 ± 1.79 and 98.02 ± 1.60 (Table S4).

### Applications of the Proposed Method

The ultra-sensitivity of the fluorimetric method was utilized for estimation of VON in spiked human plasma. The percent of recovery was found to be in the range 96.16 ± 1.21 to 98.80 ± 0.92% for the investigated method at 6 different concentration levels (Table 4).

**Table 4:** Application of the proposed method in spiked human plasma

**Added conc. (ng mL**^**−1**^**)**	% Recovery^*^ ± RSD
15	96.02 ± 1.20
50	97.10 ± 1.40
100	98.00 ± 1.52
130	97.41 ± 1.71
150	96.45 ± 1.60
200	97.30 ± 1.08

^*^Average of six determinations

In addition to, VON was determined in real human plasma (pharmacokinetic study) using 6 healthy human volunteers, VON concentration was calculated in the plasma samples after time intervals (0.25, 0.5, 0.75, 1, 1.5, 2, 3, 4, 6, 10, 15, 20 and 24 h).

The peak plasma level (C max) of cited drug was found to be 71.03 ± 1.12 ng mL^−1^ after administrated single oral dose 40 mg/tablet. The maximum time for absorption was found (t_max_) 1.5 h. with AUC equal to 429.45 ± 55.45 ng h. mL^−1^ (Fig. S4).

The results of the proposed method agrees with previously reported method [18] which refer to high sensitivity and good reliability of the proposed method.

*Besides,* the proposed method was successfully applied for determination of VON in pharmaceutical dosage form, and the obtained results were found to be satisfactory with a good recovery (102.40 ± 0.70). Using *student`s t*-test (1.40) and *F*-test (2.65) with respect to accuracy and precision and showed no significant difference between the calculated and theoretical values of both the proposed and the reported method [5] at 95% confidence level.

To ensure the consistency of dosage units, each unit in a batch should have a drug substance content within a narrow range around the label claim. For VON, due to the high sensitivity of the proposed method and its ability to rapidly measure the fluorescence intensity of a single tablet extract with sufficient accuracy, the method was ideally suited for content uniformity testing which is a time-saving process when using other conventional assay techniques. The steps of the test were adopted according to the USP procedure [3, 12]. The content of individual dosage form was analyzed. The acceptance value (AV) was calculated, and it was found to be smaller than the maximum allowed acceptance value (L1). The results demonstrated an excellent drug uniformity as shown in Table 5.

**Table 5:** Content uniformity for determination of VON using the proposed method

	**(vonaspire®** **tablets 40 mg)**
1	99.81
2	99.95
3	100.19
4	99.77
5	100.62
6	98.90
7	101.01
8	100.60
9	98.63
10	101.02
Mean	100.05
SD	0.66
RSD	0.65
Acceptance value (AV)*	1.58
Max. allowed AV (L1)*	15

^*^Acceptance value = 2.4 × SD

## Conclusion

Eventually, as far as we know, this is the most sensitive (LOQ 8.50 ng mL^−1^) and selective method for rapid and economic estimation of VON in real human plasm and in formulations using NBD-Cl reagent in alkaline medium (pH 8.2). The method was applied in pharmacokinetic study with maximum plasma concentration equal to 71.03 ± 1.12 ng mL^−1^. The presented study easily applied in clinical laboratories and therapeutic drug monitoring system.

## Supplementary Information

Below is the link to the electronic supplementary material.

Supplementary file1 (DOCX 60 kb)

## Data Availability

All data generated or analyzed during this study are included in this published article (and its supplementary information files).

## Abbreviations

**NBD-Cl**: 7-Chloro-4-nitrobenz-2-oxa-1,3-diazole
**VON**: Vonoprazan
**PK**: Pharmacokinetic
**AUC**: Area under curve
