# Supplementary material for: Ultra-Sensitive Fluorimetric Method for the First Estimation of Vonoprazan in Real Human Plasma and Content Uniformity Test
Source: J Fluoresc. 2022 Jun 7;32(5):1725–32. doi: 10.1007/s10895-022-02979-2 (PMC9402479; doi:10.1007/s10895-022-02979-2)
Supplement: Supplementary file 1 — Supplementary file1 (DOCX 60 kb) [file 10895_2022_2979_MOESM1_ESM.docx]

**Ultra-sensitive fluorimetric method for the first estimation of Vonoprazan in real human plasma and content uniformity test**

Roshdy E. Saraya ^a,^ Yasser F. Hassan ^b^, Walid E Eltukhi ^b^& Baher I. Salman ^b*^

^a^ Pharmaceutical Analytical Chemistry Department, Faculty of Pharmacy, Port Said University, Port Said 42511, Egypt.

# ^b^ Pharmaceutical Analytical Chemistry Department, Faculty of Pharmacy, Al-Azhar University-Assiut branch-Assiut 71524, Egypt. [*bahersalman@azhar.edu.eg*](mailto:bahersalman@azhar.edu.eg)


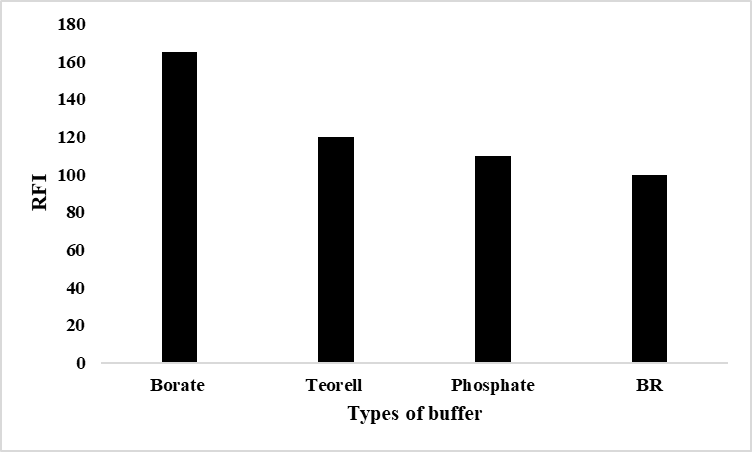


**Fig. S1** Effect of type of buffers for reaction of VON with NBD-Cl.


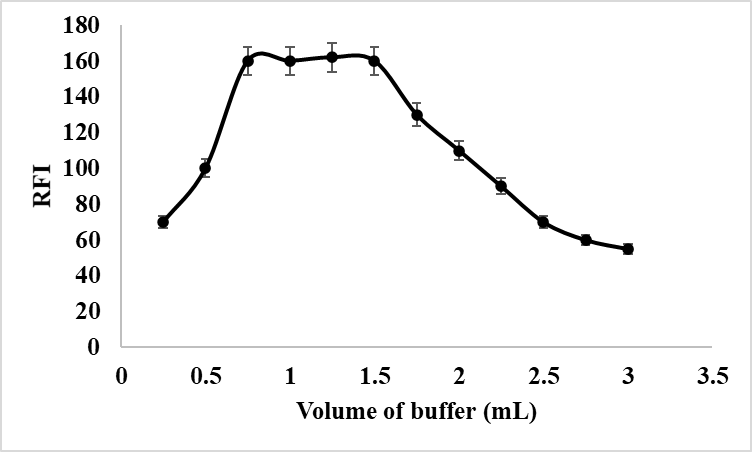


**Fig. S2** Effect of volume of buffer for reaction of VON with NBD-Cl.


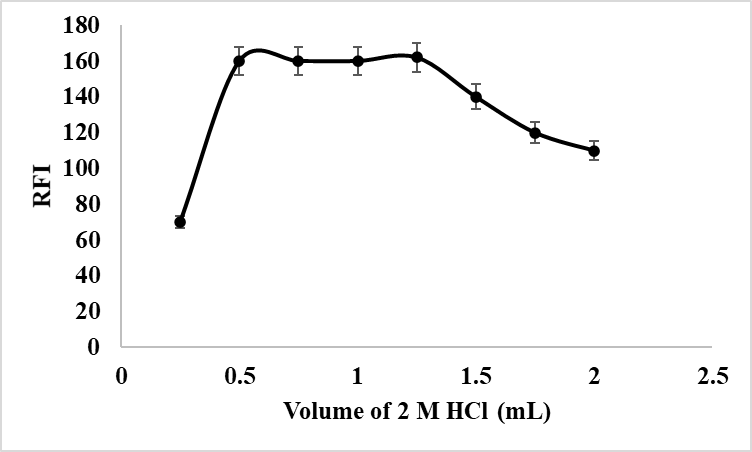


**Fig. S3** Effect of volume of 2M HCl for reaction of VON with NBD-Cl.

**Table S1** Robustness of the proposed method

|  | | **%Recovery^*^** | **%RSD** | | |  |
| --- | --- | --- | --- | --- | --- | --- |
| **1- Effect of pH (Borate buffer)** | | | | |  |  |
| **8.0** | 99.86 | | | 0.18 | |  |
| **8.4** | 99.71 | | | 0.35 | |  |
| **2- Volume of borate buffer (pH 8.2) mL** | | | | |  |  |
| **0.75** | 99.90 | | | 1.00 | |  |
| **1.25** | 99.00 | | | 0.66 | |  |
| **3- Volume of NBD-Cl, mL** | | | | |  |  |
| **0.4** | 99.56 | | | 0.44 | |  |
| **0.6** | 99.23 | | | 0.26 | |  |
| **4– Temperature, ^o^C** | | | | |  |  |
| **75** | 99.40 | | | 0.60 | |  |
| **90** | 99.59 | | | 0.71 | |  |
| **5- Heating time, min** | | | | |  |  |
| **12** | 99.44 | | | 0.90 | |  |
| **17** | 99.70 | | | 0.95 | |  |
| **6- Volume of 2 M HCl, mL** | | | | |  |  |
| **0.5** | 99.90 | | | 0.99 | |  |
| **1.00** | 99.88 | | | 0.51 | |  |

**^*^:** Mean of six determinations.

**Table S2** Incurred sample reanalysis for estimation of VON using the proposed method

| **Sample** | **Intial concentration* (ng mL^-1^) ± SD** | **Incurred concentration***  **(ng mL^-1^) ± SD** | **% Deviation** |
| --- | --- | --- | --- |
| **1** | 71.50 ± 0.99 | 68.95 ± 2.01 | - 3.56 |
| **2** | 70.90 ± 1.87 | 68.04 ± 1.58 | - 4.03 |
| **3** | 71.10 ± 1.60 | 69.80 ± 1.69 | - 1.82 |

*: Mean of three determinations

**Table S3.** Selectivity of the proposed methods

|  | **Recovery* ± RSD** |
| --- | --- |
| **Mannitol (10 mg)** | 100.22 ± 0.32 |
| **Talc (10 mg)** | 101.02 ± 0.55 |
| **Starch (100 mg)** | 100.31 ± 0.61 |
| **Lactose (10 mg)** | 100.40 ± 0.80 |
| **Magnesium stearate (10 mg)** | 100.21 ± 0.43 |
| **Sodium chloride (10 mg)** | 99.09 ± 0.65 |

*: Mean of three determinations

**Table 5** Stability of VON in human plasma under different conditions.

| **Conditions** | **VON** | | |
| --- | --- | --- | --- |
| **Concentrations** | **LQC**  **20 ng mL^-1^** | **MQC**  **100 ng mL^-1^** | **HQC**  **150 ng mL^-1^** |
| Three Freeze–thaw cycle stability (-24°C) | 95.11 ± 1.79 | 96.15 ± 1.88 | 95.76 ± 1.04 |
| Long-term stability (1 month at -24°C) | 96.34 ± 1.32 | 97.06 ± 1.78 | 96.66 ± 0.88 |
| Short-term stability (12 h at -24°C) | 96.55 ± 1.05 | 97.03 ± 1.90 | 98.02 ± 1.60 |
| Post-preparative stability (6 h at room temperature 25 °C) | 95.88 ± 0.99 | 97.18 ± 1.68 | 97.25 ± 1.73 |
| Post-preparative stability (12 h at room temperature 25 °C) | 96.42 ± 0.87 | 95.89 ± 1.11 | 96.16 ± 1.52 |

Data presented as recovery (%) ±SD (n = 5).


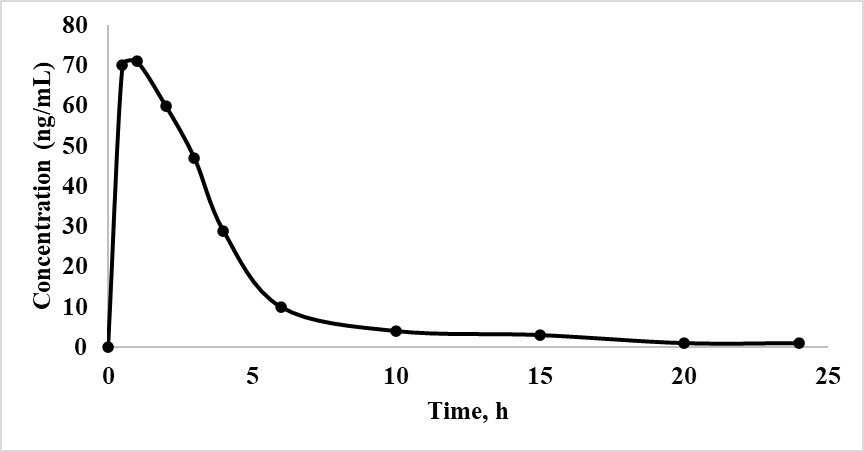


**Fig. S4** pharmacokinetic parameters of VON using the proposed method
